# A Comparative Evaluation of Conventional Instrumentation and Accelerometer-Based Navigation in the Practice of a High-Volume Unicompartmental Knee Arthroplasty (UKA) Surgeon

**DOI:** 10.1016/j.artd.2023.101272

**Published:** 2023-11-23

**Authors:** Jeremiah M. Taylor, Jay N. Patel, Christopher J. Mazzei, Adam A. Sassoon

**Affiliations:** a Department of Orthopaedic Surgery, University of California Los Angeles, Los Angeles, CA, USA; b Department of Orthopedic Surgery, Morristown Medical Center, Morristown, NJ, USA

**Keywords:** Accelerometer-based navigation, Radiographic outliers

## Abstract

**Background:**

Component malpositioning and joint malalignment following unicompartmental knee arthroplasty (UKA) increase the risk for revision. This study investigates whether accelerometer-based navigation (NAV) decreases radiographic outliers with respect to component placement and joint alignment in comparison to conventional instrumentation in UKA.

**Methods:**

A radiographic review of UKAs was performed by a single surgeon following adoption of an accelerometry-guided navigation system (OrthAlign, Aliso Viejo, CA). This cohort was then compared to previous patients undergoing UKA with conventional instrumentation. Six-week postoperative radiographs were used to compare femoral coronal and sagittal angles, tibial coronal and sagittal angles, the net coronal angle, tibial component rotation, and medial tibial overhang. Outliers in implant positioning were compared between groups. Patient variables including age, gender, body mass index, American Society of Anesthesiology, and surgical time (incision until the start of closure) were also compared between groups.

**Results:**

Eighty-eight UKA’s were reviewed (49 conventional instrumentation [CI] patients; 39 NAV patients). Using 2-sample t-tests, no significant differences were found in patient demographics, radiographic parameters, and operative times between the CI and NAV cohorts. Using chi-squared tests, no significant difference was found in the number of radiographic outliers between the CI and NAV cohorts.

**Conclusions:**

Our study found that a high-volume UKA surgeon achieved a low rate of radiographic outliers in both NAV and CI cohorts. This data suggests that NAV is no different from conventional instrumentation with respect to implant positioning, overall joint alignment, and operative time when used by a high-volume UKA surgeon.

## Introduction

Unicompartmental knee arthroplasty (UKA) is a bone-conserving and ligament-sparing procedure used to treat patients with localized osteoarthritis, most commonly in the medial compartment. The advantages of UKA procedures are shorter recovery, improved kinematics, better functional outcomes, and lower cost compared to total knee arthroplasty (TKA) [[1], [2], [3]]. These advantages have resulted in increased usage of UKA over the past 2 decades [4]. Currently, UKA accounts for 5%-11% of all knee arthroplasties worldwide and is expected to have a 6-fold increase in utilization by 2030 [[5], [6], [7], [8], [9], [10], [11]].

Despite the advantages over TKA, several studies have questioned the long-term survivorship of UKA. Recent literature has shown UKA to have a revision rate of 4.9%-14.2% at a mean of 3-3.7 years [12,13]. A significant source of early implant failure is component malalignment and overhang. Compared to implants with no alignment or overhang errors, those with errors have a 15.2%-17.9% higher failure rate [12]. The technical surgical errors are secondary to varying surgeon experience, as Baker et al. [14] showcased a decreased implant failure rate in surgeons performing >15 UKAs per year. Periprosthetic fractures represent another mode of failure. Although this complication has an incidence rate of 0.6%-1%, it often leads to adverse outcomes, including the need for conversion to TKA [15,16]. Patient characteristics such as younger age and higher body mass index (BMI) are associated with an increased risk of UKA failure [17,18]. The increasing rates of UKA performance highlight the need to better understand and mitigate these risk factors to better optimize patient outcomes and lower revision rates.

Handheld accelerometer-based navigation (NAV) has been developed to improve the accuracy and precision of UKA component position and alignment. Many reports have shown these systems are reliable in improving implant position in TKA without increasing the operative time [[19], [20], [21], [22]]. However, there is limited data on NAV outcomes in UKA. The primary aim of this study was to determine if the use of NAV instrumentation in UKA provides greater accuracy and implant positioning. The secondary aim was to assess the difference in surgical time between UKA procedures utilizing conventional instrumentation (CI) and NAV.

## Material and methods

A radiographic review of UKAs was performed by a single surgeon following adoption of an accelerometry-guided navigation system (OrthAlign, Aliso Viejo, CA). In this study, 88 consecutive patients who underwent primary medial fixed-bearing UKA between September 2018 and March 2021 were included. The first 43 patients underwent UKA with CI. There was a temporal point in which NAV technology was introduced to the senior surgeon. Following this, only 6 patients did not undergo UKA with NAV. In these instances, the NAV operative trays were not available. There were no instances in which NAV was aborted in favor of CI. The CI cohort consisted of 49 patients, while the NAV cohort consisted of 39 patients.

All patients undergoing UKAs with CI underwent a midvastus approach to the knee. The sagittal contact axis of the femur and tibia was marked to establish the rotation of the tibial resection. An extramedullary cutting guide was applied to the operative extremity to determine the coronal angulation and posterior slope of the tibial resection with the goals of cutting the tibia in a coronal mechanically neutral position and 6 degrees of posterior slope. The resection depth was set at 4 mm from the lowest point of the medial tibial plateau. Following the tibial resection, flexion and extension gaps were balanced. When the extension gap was found to be comparatively lax, the distal femoral resection was performed in an appropriately conservative position. The femur was then sized, and the appropriate cutting block was applied to the femur for performance of posterior and chamfer cuts. Meniscal remnants and posterior osteophytes were then resected. We subsequently sized the tibial component and impacted the trial components in place. All knees were able to achieve full extension without hyperextension, flexion to 120 degrees with gravity, and were noted to be stable with varus/valgus stress testing at full extension at 30 degrees and 90 degrees of flexion. We also noted appropriate patellar tracking in each case. Once satisfied with the trial components, we packed the knee with lap sponges, exsanguinated the limb using an Esmarch, and inflated the tourniquet to 250 mmHg for purposes of cementation. We removed the trial components and cemented the real components in place. Once the real components were cemented in place, the limb was positioned in full extension while an axial load was applied during the cement curing process. Once the cement had cured, we explored the joint for any excess remaining cement, which was removed meticulously. We then copiously irrigated the knee with 3 L of sterile saline and performed a layered closure.

**Figure 1:**
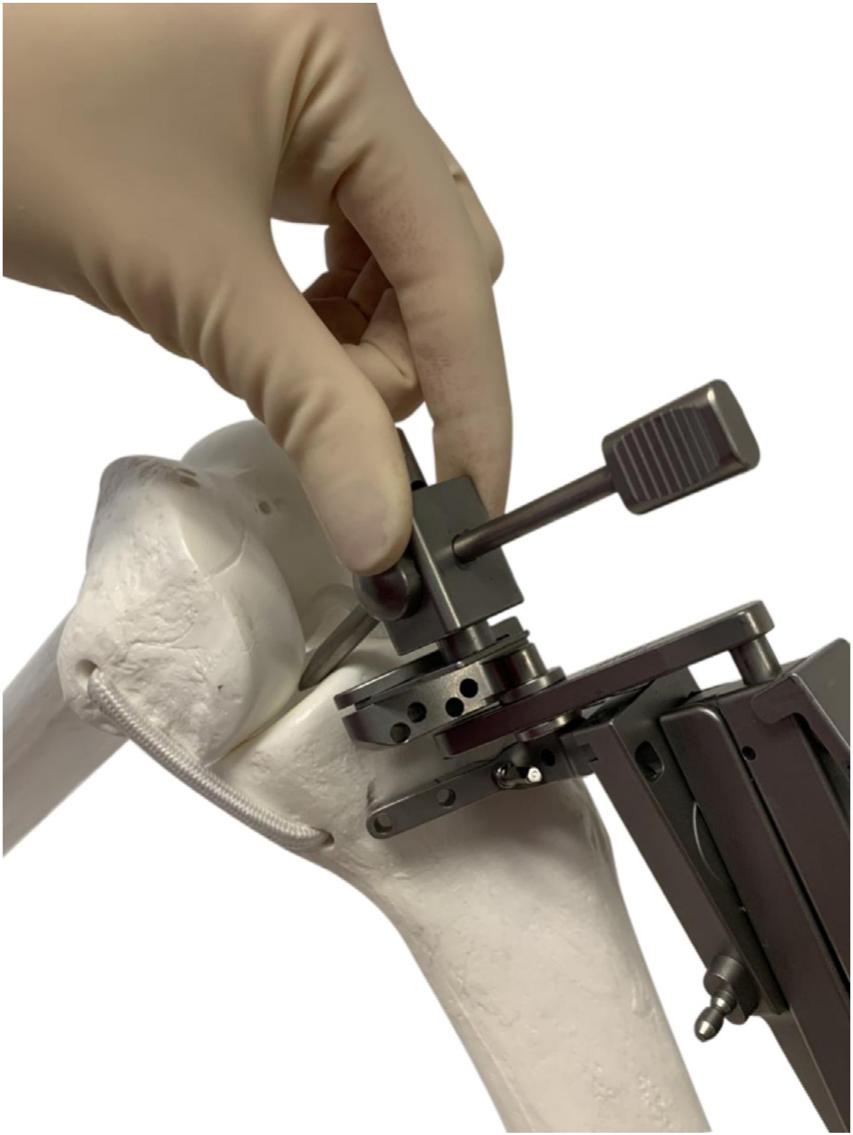
A schematic illustrating the depth perception process prior to tibial cutting in a skeletal model. Courtesy of OrthAlign Corporation, 120 Columbia, Suite 500, Aliso Viejo, CA 92656, USA.

**Figure 2:**
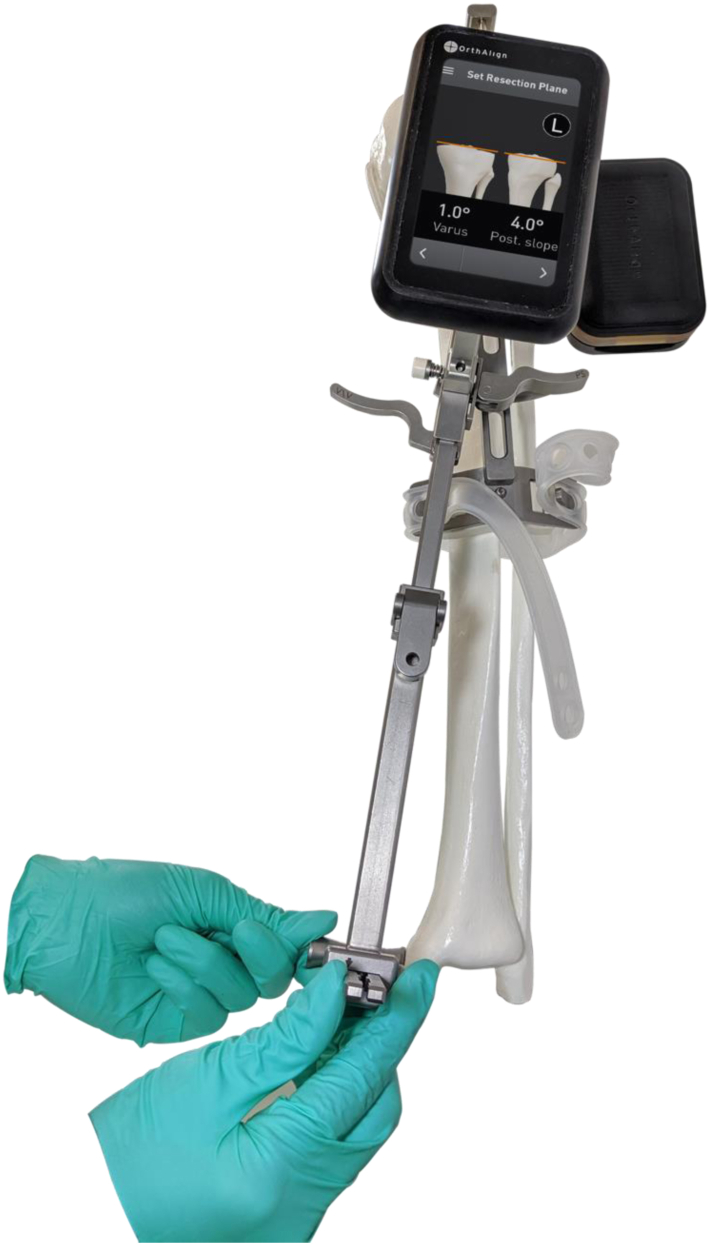
A precise depiction of the alignment process for the tibial cutting angle with respect to the medial malleolus on a skeletal model. Courtesy of OrthAlign Corporation, 120 Columbia, Suite 500, Aliso Viejo, CA 92656, USA.

**Figure 3:**
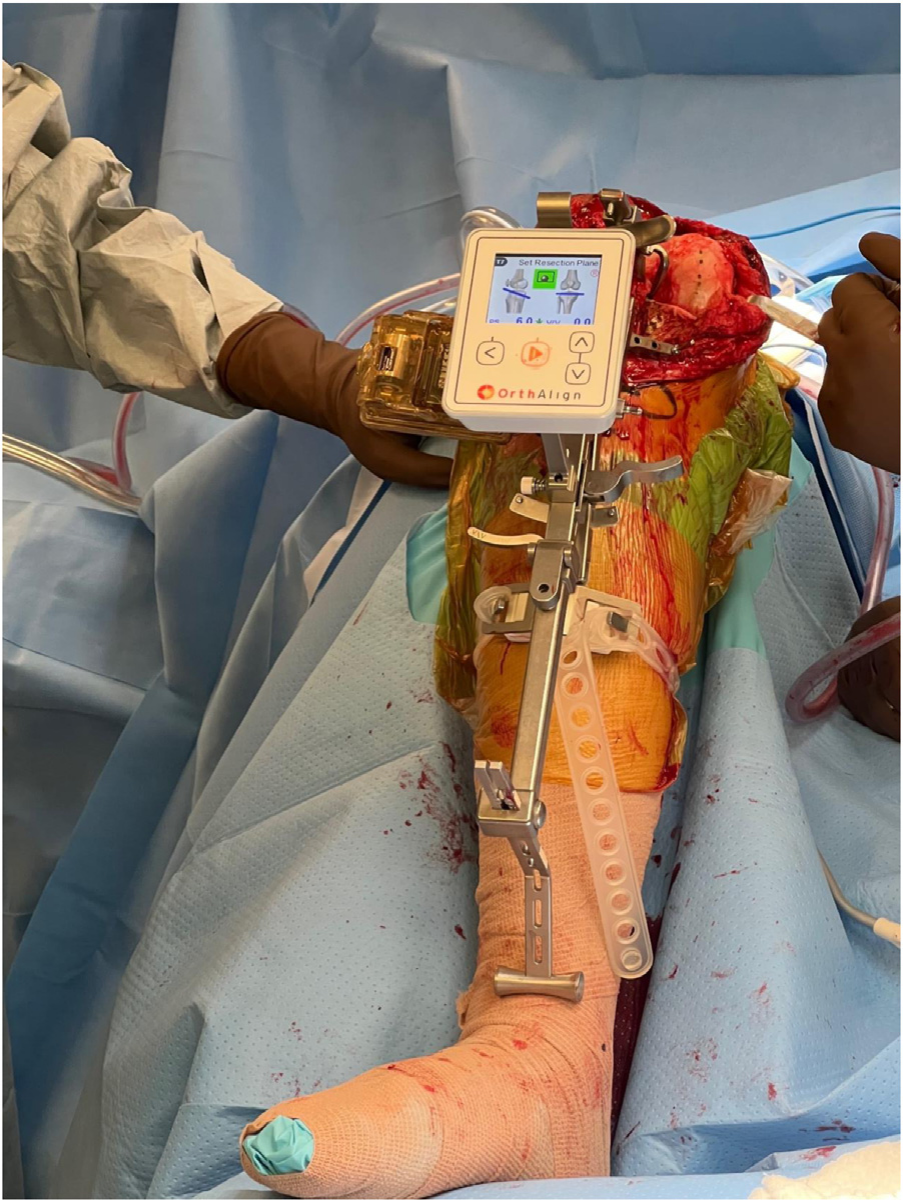
Intraoperative photograph illustrating the utilization of accelerometer-based navigation during a unicompartmental knee arthroplasty case.

For all UKAs with NAV, once the anticipated rotational axis of the tibial resection was marked, we mounted the OrthAlign (AlisoViejo, CA) tibial cutting guide to the operative extremity using an elastic strap and a single-headed pin. We then attached the sensors to the cutting guide and registered the offset of the guide relative to the anterior cruciate ligament footprint as well as the medial and lateral malleoli to navigate our tibial resection in 6 degrees of posterior slope with a neutral coronal axis relative to the mechanical axis of the tibia. We set our resection depth at 4 mm off the tibial plateau and performed our resection (Figure 1, Figure 2, Figure 3). The remaining steps of the procedure were identical. All 88 patients received a Journey Oxinium Surface Femur Prosthesis by Smith and Nephew (Memphis, TN), as well as a Zuk all-polyethylene tibial component, also manufactured by Smith and Nephew. Of note, the latter product was originally manufactured by Zimmer before being acquired by Smith and Nephew in June 2015.

**Figure 4:**
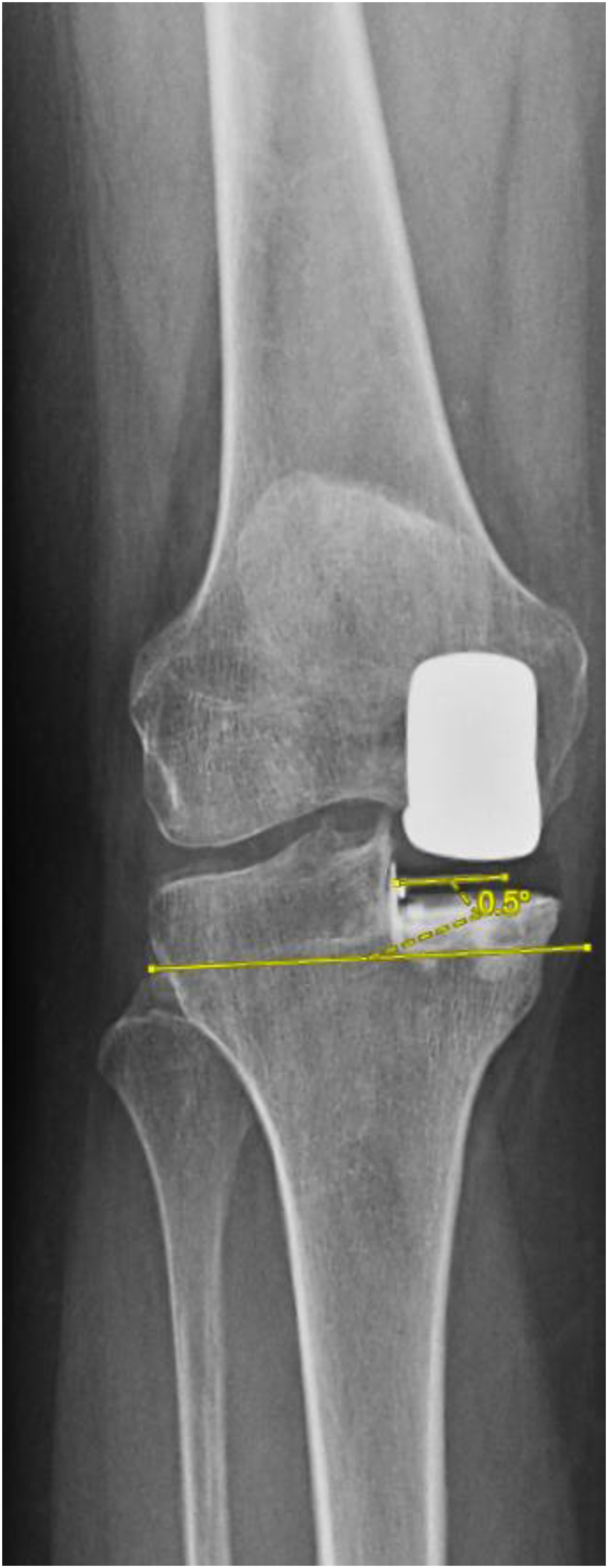
Example TCA = 0.5° varus.

Routine short-leg, weight-bearing radiographs obtained at the 6-week postoperative time point were analyzed using Materialise OrthoView (Meridian Technique Limited; Chilworth, Hampshire, United Kingdom) to measure femoral coronal and sagittal angles (FCA and FSA), tibial coronal and sagittal angles (TCA and TSA), tibial component rotation (TCR), and medial tibial overhang (MTO), Figure 4. The net coronal angle (NCA) was calculated by adding FCA and TCA. All UKAs were performed using a standard midvastus approach. No custom cutting guides or fluoroscopy was used. The surgical time was measured as the time from incision to the start of wound closure.

Patient demographics such as BMI, American Society of Anesthesiology scores, age, and gender were also collected, Table 1. UKA inclusion criteria included advanced osteoarthritis isolated to the medial compartment, the presence of a functional anterior cruciate ligament, noninflammatory arthropathy, flexion contracture <10°, fixed varus deformity <15°, and arc >90°. Of note, age and BMI thresholds were not utilized for study inclusion. All surgeries were performed by the senior author (A.A.S.), who is a fellowship-trained arthroplasty surgeon. During the study period, UKA accounted for approximately 40% of his overall primary knee arthroplasty volume. Statistical analysis was performed using Minitab (State College, PA). Radiographic outliers were calculated as previously defined in the literature [12]. This includes FCA > ±10° deviation from 0°, FSA >15° flexion, TCA > ±5° deviation from 0°, TSA >±5° deviation from 7°, and MTO >2 mm, Table 3.

**Table 1:** Statistical analysis of patient characteristics and surgical variables.

Patient demographics	NAV	CI	*P*-value
Age (y)	64.6	66.2	.341
Patient total	39	49	
Body mass index (BMI)	30.65	30.26	.717
ASA	2	2	.454
% Male	58.97%	38.78%	
% Female	41.03%	61.22%	
Surgical variables			
Surgical time (min)	54.4	54.1	.919

ASA, American Society of Anesthesiology.

**Figure 5:**
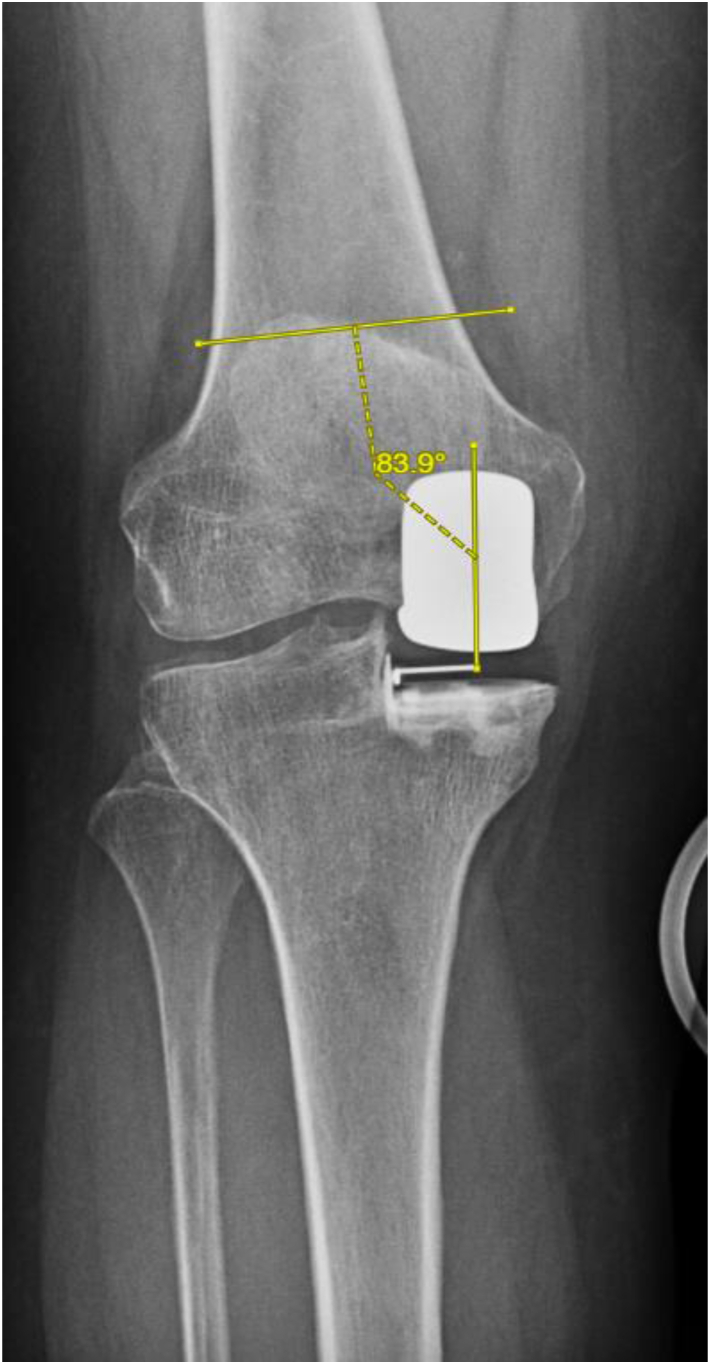
Example FCA = 6.1° valgus.

**Figure 6:**
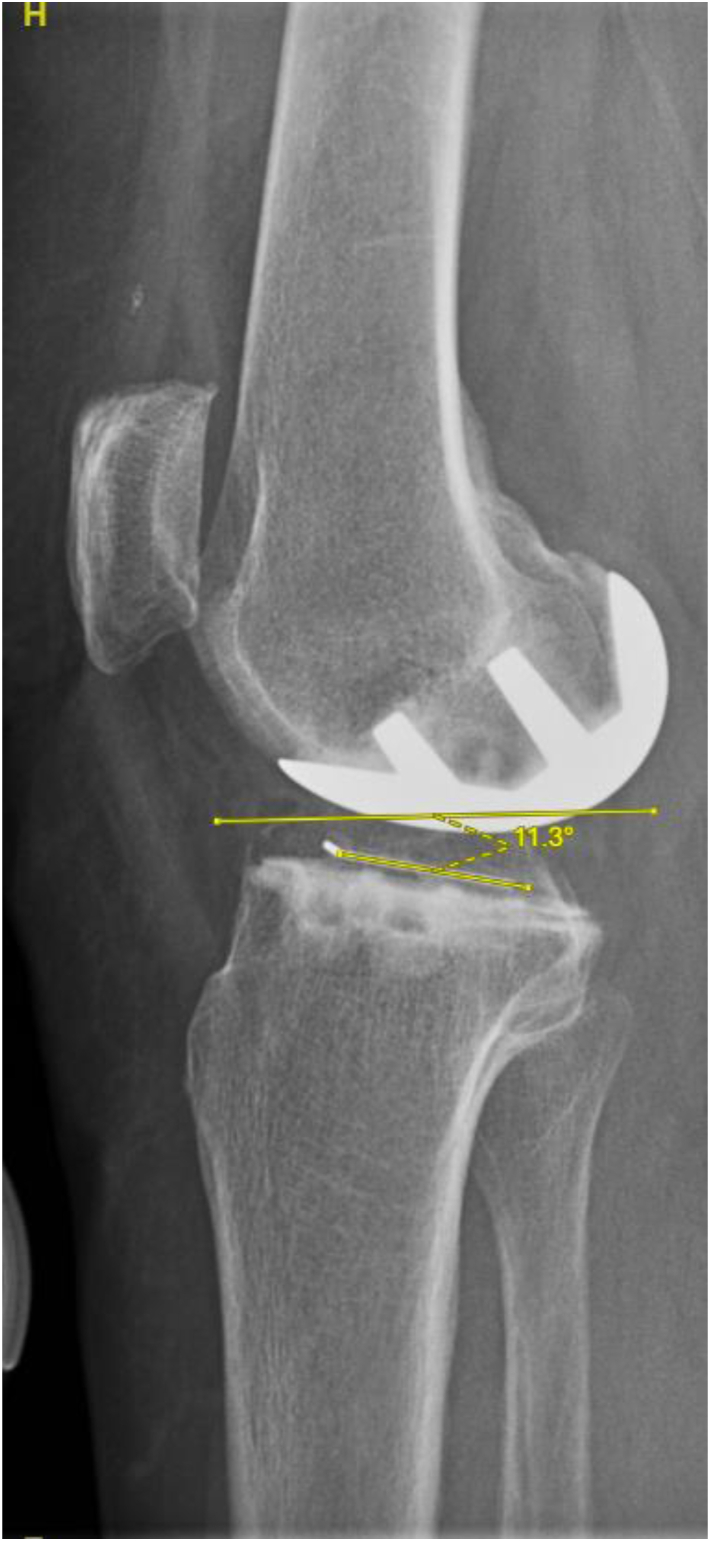
Example TSA = 11.3° posterior slope.

**Figure 7:**
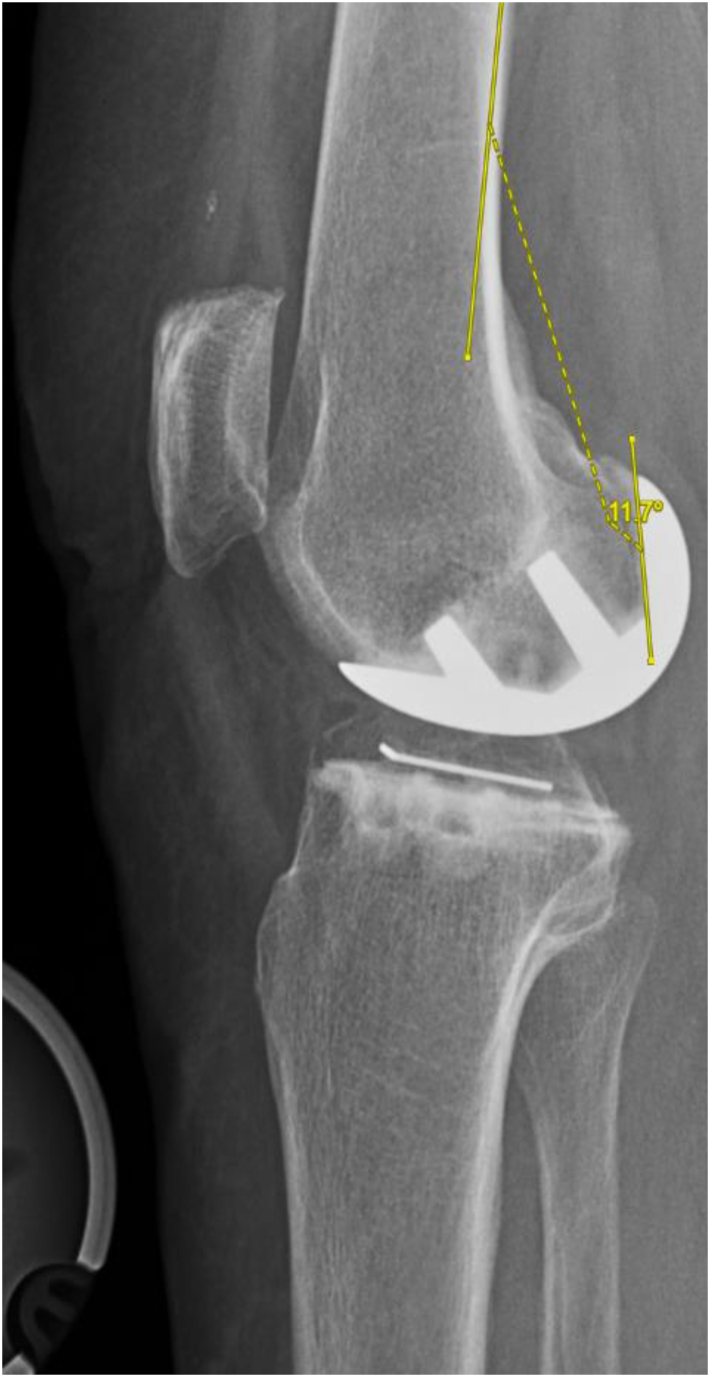
Example FSA = 11.7° flexion.

**Figure 8:**
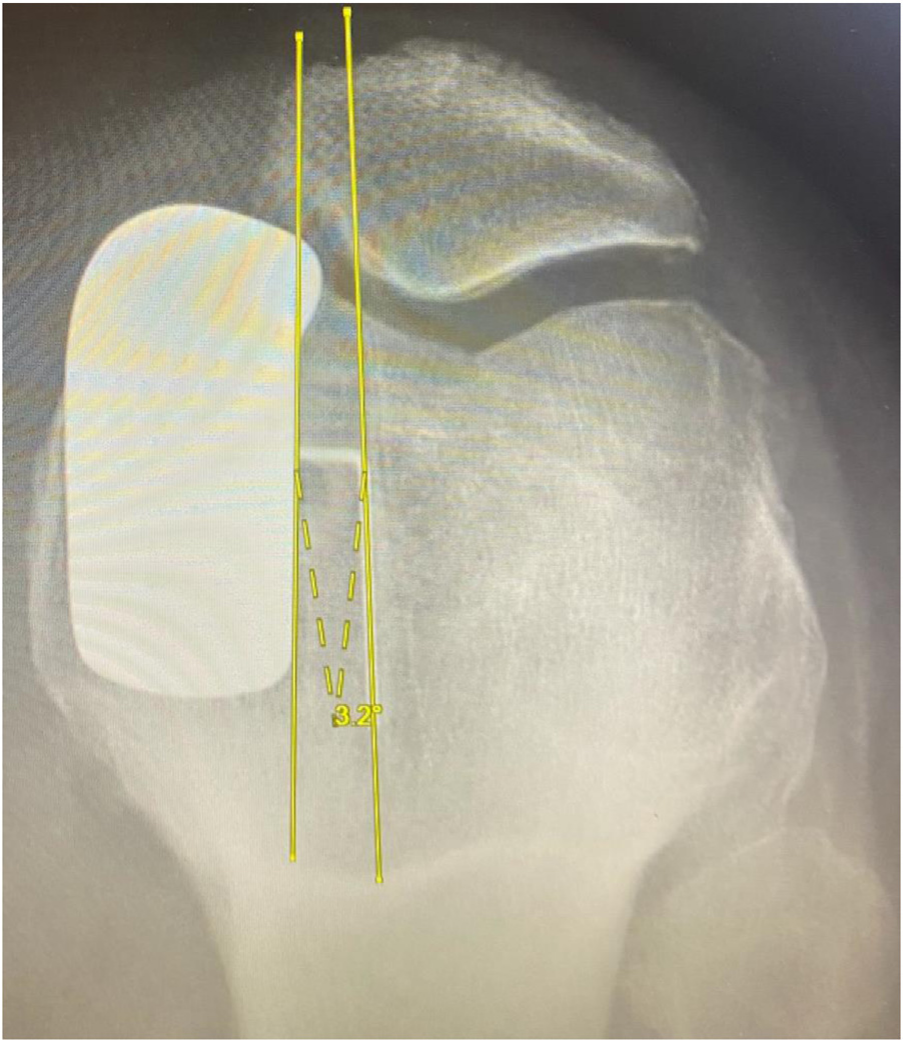
Example TCR = 3.2° internal rotation.

To acquire the angle measurements, the following approach was utilized in Materialise OrthoView. For the TCA, a line parallel to the anatomic axis of the tibia was drawn. A second line was drawn perpendicular to the first. Then, the component angle was derived from the degrees of angulation between the tibial component plateau and the second reference line, Figure 4. For the FCA, a line parallel to the anatomic axis of the femur was drawn. A second line perpendicular to the first was then drawn. Following this, a line bisecting the femoral component was drawn, and the angle was derived from the degrees between the bisected femoral line and second reference line, Figure 5. For the tibial sagittal angle, a line bisecting the tibia was drawn. Following this, a line perpendicular to the first was made. The angle was deciphered as the slope between the tibial component and the second line, Figure 6. For the femoral sagittal angle, a line along the posterior cortex of the femur was drawn, and a second line tracing the posterior portion of the femoral component was made. The resulting angle of these 2 lines was utilized as the FSA, Figure 7. For the TCR angle, sunrise-view radiographs were utilized. A line tracing the lateral border of the femoral component and a line tracing the implant pin were drawn. The angle between these 2 lines was utilized as the TCR, Figure 8.

### Statistical methods

Our data was analyzed using Statistical Package for Social Sciences (SPSS) (IBM Corp., Armonk, NY, USA). Patient demographics, radiographic parameters, and operative times were compared between the CI and NAV groups using independent sample t-tests for continuous variables and chi-square tests for categorical variables. The primary outcome measures were FCA, FSA, TCA, TSA, NCA, TCR, and MTO. The secondary outcome measure was the surgical time (incision until the start of closure). The number of radiographic outliers between the CI and NAV groups was compared using chi-square tests. A *P*-value of less than .05 was considered statistically significant. All tests were 2-tailed.

## Results

A total of 88 UKAs met the inclusion criteria (49 CI; 39 NAV). Demographic variables were as follows: BMI (CI 30.26, NAV 30.65), American Society of Anesthesiology (CI 2, NAV 2), age (CI 66.2, NAV 64.6), and gender (CI 38.78% male, 61.22% female; NAV 58.97% male, 41.03% female). There were no significant differences in demographic variables between the 2 cohorts, Table 1. Analysis with a 2-sample t-test showcased no significant difference between the surgical times of the cohorts (NAV 54.4 minutes; CI 54.1 minutes), Table 1.

Using 2-sample t-tests, no significant difference was found in FCA (1.13° valgus, 1.23° valgus; *P* = .894), FSA (12.88° flexion, 13.26° flexion; *P* = .632), TCA (0.66° varus, 0.04° valgus; *P* = .206), TSA (6.27° posterior slope, 7.14° posterior slope; *P* = .211), NCA (0.47° valgus, 1.27° valgus; *P* = .377), TCR (0.28° internal rotation, 1.43° internal rotation; *P* = .476), and MTO (0.04 mm, 0.13 mm; *P* = .204) between the CI and NAV cohorts, respectively; Table 2. Using chi-squared tests, no significant difference was found in the number of outliers amongst FCA (0, 0), FSA (10, 13; *P* = .170), TCA (2, 3; *P* = .467), TSA (6, 1; *P* = .095), and MTO (0, 1) between the CI and NAV cohorts, respectively; Table 3.

**Table 2:** Radiographic outcomes.

Measurement	NAV	CI	*P*-value
Femoral coronal angle (FCA)	1.23^○^ Valgus	1.13^○^ Valgus	.894
Femoral sagittal angle (FSA)	13.26^○^ Flexion	12.88^○^ Flexion	.632
Tibial coronal angle (TCA)	0.04^○^ Valgus	0.66^○^ Varus	.206
Tibial sagittal angle (TSA)	7.14^○^ Posterior slope	6.27^○^ Posterior slope	.211
Net coronal angle (NCA)	1.27^○^ Valgus	0.47^○^ Valgus	.377
Tibial component rotation (TCR)	1.43^○^ Internal rotation	0.28^○^ Internal rotation	.476
Medial tibial overhang (MTO)	0.13 mm	0.04 mm	.204

**Table 3:** Radiographic outliers.

Measurement	NAV	CI	*P*-value
Femoral sagittal angle (FSA)	13	10	.170
Femoral coronal angle (FCA)	0	0	
Tibial sagittal angle (TSA)	1	6	.095
Tibial coronal angle (TCA)	3	2	.467
Medial tibial overhang (MTO)	1	0	
Met all 4 angle alignment targets	23	34	.309

Radiographic outlier definitions. FCA: >±10^○^ deviation from 0^○^; FSA: >±15^○^ of Flexion; TCA: >±5^○^ deviation from 0^○^; TSA: >±5^○^ deviation from 7^○^; MTO >2 mm.

We observed a trend in the CI cohort demonstrating a greater incidence of TSA outliers (6, 1); however, this trend failed to achieve statistical significance. In the CI cohort, the 6 TSA outliers consisted of 2 instances where the TSA exceeded a deviation of +5° from the set standard of 7° (resulting in >12°) and 4 instances where the TSA showed a deviation of less than −5° from the standard of 7° (resulting in <2°). The absolute values corresponding to these 6 instances were −7°, −4°, −3.5°, 1.5, 12.5°, and 12.5°. In contrast, the sole NAV TSA outlier exhibited an absolute value of 12.6°. All outlier definitions were based on previous literature published by Kazarian et al. [12].

## Discussion

This data suggests that NAV in the hands of a high-volume UKA surgeon is equivalent to CI in respect to implant positioning, overall joint alignment, and operative time. To our knowledge, this study represents the only comparison of NAV to CI in UKA. Moreover, our data showcased a higher accuracy in both cohorts than previously published work, potentially explained by the senior surgeons’ fellowship training and high-volume UKA caseload.

Previous literature has shown that radiographic outliers correlate with an increased risk of UKA failure. For both groups, the radiographic outlier frequency was lower than previous literature, but no differences existed between groups [12]. In previous studies, surgeons performing an average of 14 UKAs per year met all 4 alignment measures (FCA, FSA, TCA, and TSA) in 19.0% of cases [12]. Our CI and NAV cohorts met all alignment measures in 69.3% and 58.9% of cases, respectively; Table 3. This represents substantial improvement compared to previous literature, further highlighting the importance of surgeon volume and NAVs potential role to assist in surgical precision. Through our comparison, both cohorts showcased similar FCA, FSA, TCA, TSA, NCA, TCR, and MTO radiographic measurements; Table 2.

At the onset of the study, NAV was a novel technology for the senior surgeon in the setting of UKA. However, the surgeon had utilized this system regularly for performance of TKAs in his practice with almost identical instrumentation for the navigated tibial resection. Consequently, this resulted in a virtually absent learning curve. This was evidenced by the fact that there was no increase in operative times, no instances where NAV was abandoned in favor of CI use, and no cases had to be excluded from the dataset due to issues with this new technology. This is parallel to the rapid learning curve seen in other studies [23].

Notably, the operative time was not adversely affected by NAV usage (CI 54.1 minutes, NAV 54.4 minutes, *P* = .919), a finding on par with a recently published multicenter randomized control trial [24]. The similarity in operative times between the CI and NAV groups can be reasonably attributed to the user-friendly design of the NAV system. As a single-hand unit, NAV allows surgeons to set the coronal alignment, sagittal alignment, resection depth, and resection rotation in a stepwise progression. This obviates the need to hold extramedullary cutting guide in 3 planes simultaneously, thereby facilitating the ease of performing the tibial resection. These findings are a stark contrast with the literature published on large-console computer-assisted surgical navigation systems (LCNS), which showcase operative times upwards of 130 minutes [25]. Taken together, this data indicates that in the hands of a high-volume UKA surgeon, NAV is no different from CI and does not increase operative time.

There were several limitations to our study. This includes the temporal bias present between the 2 groups. Due to the NAV procedures occurring in succession to the CI procedures, we recognize there was an opportunity for the senior surgeon to correct inadequate bone cuts made with NAV. However, there was no instance in which NAV was aborted in favor of CI, and no bony recuts were performed. Following the technique divergence point, CI was utilized in 6 procedures where NAV trays were not available in the operating room. Secondly, all radiographic angles were performed on short-leg, weight-bearing 6-week postoperative radiographs, while each radiograph was standardized and reviewed at the time of the patient’s 6-week appointment for quality, small differences in patient positioning can lead to errors in angle measurements. Though the angles were measured in Materialise OrthoView software, small differences in user measurements are likely. All radiographic measurements were done by at least 2 authors using the same techniques to ensure precision. Lastly, though we had 88 total patients, increased patient numbers and randomization in each cohort would help increase the overall power of the study and decrease any potential biases, respectively.

Current literature has analyzed the difference between LCNS and CI. We understand this was not a comparative study between LCNS and NAV. However, our data showcases NAV as a promising alternative.

LCNS presents several disadvantages including cost, increased operative time, line-of-sight issues, closed platform software that limits surgical implant choices, and system-specific training of surgeons and staff.

NAV systems offer advantages and solutions to some of the utilization drawbacks of LCNS. NAV systems require no large capital purchases, no requirement for preoperative advanced imaging, no additional pin tracks, and no potential complications from pin site infections and/or fractures. In addition, NAV benefits from providing a more similar feel and operative flow to conventional intra-/extra-medullary alignment jigs and an operative time similar to CI usage. Limitations to NAV include an inability to quantitatively assess soft tissue balancing, lack of information regarding femoral and tibial rotation, the cost associated with disposable single-use NAV system parts, and the potential for imprecise calibrations [26]. More specifically, the NAV device utilizes the lateral and medial malleoli as calibration landmarks when deciphering the appropriate tibial cutting angle. This may affect the precision and accuracy of the device when utilized on patients with excess soft tissue near the malleoli as well as those with ankle deformities. This may have played a role in the prevalence of outliers within the NAV cohort, as all NAV patients with outliers in TSA (n = 1) and TCA (n = 3) possessed BMIs greater than 31. Special care should be taken by surgeons when utilizing this device in those with excess soft tissue and ankle deformities to ensure the malleoli are manually palpated and the device is placed precisely over these anatomic landmarks during the calibration phase.

NAV has been widely studied in TKA. Nam et al. assessed 80 TKAs performed with NAV compared to a matched cohort of 80 TKAs performed with a large-console, imageless computer assisted surgery (CAS) system. Postoperative component and overall alignment were found to be as accurate and precise between the NAV and large console, imageless CAS systems [27]. The same authors then performed a randomized controlled trial to compare tibial component alignment following TKA utilizing either NAV or extramedullary conventional guides. Standing anterior/posterior hip-to-ankle and lateral knee-to-ankle radiographs were analyzed in 94 patients. They found NAV to be statistically superior in the accuracy and precision of tibial alignment, with 95.7% of TKA from the NAV group being <2° from the neutral mechanical axis compared to 68.1% from the conventional cohort (*P* < .001). Evaluation of the tibial component slope demonstrated similar results with 95.0% of NAV patients <2° from the 3° posterior slope goal, compared to 72.1% of conventional cohort patients (*P* = .007) [19]. These studies illustrate NAVs potential to enhance surgical outcomes similar to large console, CAS systems without the capital equipment setup costs, annual system update fees, and operating room line-of-sight issues. Further research is needed to quantify NAVs benefit to UKA appropriately.

Postoperative outcomes following UKA are commonly compared to TKA. Through analysis of Knee Society Scores in a matched comparison, recent literature has shown UKA to have improved function at the 6-week and 2-year postoperative time points [28,29]. In addition, patients have less postoperative pain, earlier returns to work, and less joint awareness following UKA procedures [29]. Long-term outcome comparisons at the 5-year and 10-year time points indicate similar patient satisfaction reported by Knee Society Scores and Oxford Knee Scores [[29], [30], [31]]. Despite similar functional outcomes, the long-term survival rate of UKA has been reported to be inferior to TKA [32,33]. Studies have demonstrated aseptic loosening and implant malalignment to be 2 of the more common reasons for this [34,35]. These technical surgical errors are secondary to varying surgeon UKA volume. Baker et al. [14] showed a revision rate of 2% for surgeons performing more than 100 UKAs over their 8-year study period. Surgeons with less than 25 UKAs over the same period had a 5% revision rate. In a retrospective study of 40,522 UKAs, Mohammed et al. [36] showcased superior results for high-volume surgeons during a 10-year follow-up. Surgeons who performed more than 30 cemented UKAs per year had a survival rate of 97.5% compared to 86.8% for surgeons performing less than 10 cemented UKAs per year. Furthermore, a similar pattern can be seen with respect to hospital volume. An analysis of the German general regional health insurance registry found a 5-year UKA survival rate of 84.1% in centers that performed less than 12 UKAs per year [37]. In contrast, hospitals with more than 104 UKAs per year had a 93.2% survival rate. Murray et al. [38] recommend that surgeons should perform at least 12 UKAs per year to significantly decrease the revision rate. Other studies have produced similar conclusions with a recommended threshold of 13 UKAs per year [11]. However, as Klasen et al. [39] point out, these recommendations are not feasible for most surgeons. In this context, NAV can be used to help lower-volume UKA surgeons improve component positioning and alignment, as this may improve implant survivorship.

## Conclusions

Our study found that a high-volume UKA surgeon achieved a low rate of radiographic outliers in both NAV and CI cohorts. This data demonstrates superior performance in comparison to previously reported rates of radiographic outliers. Furthermore, NAV did not contribute to an increased operative time. Our data suggests that NAV in the hands of a high-volume UKA surgeon is no different from CI in respect to implant positioning, overall joint alignment, and operative time. In addition, NAV systems have no capital costs, no requirement for preoperative advanced imaging, and no potential for pin tract infection, which contrasts with large console robotics. To our knowledge, this study represents the only comparison of NAV to CI. Larger, randomized controlled trials are needed to help further validate our results. Additionally, research involving NAV use in low-volume or less experienced UKA surgeons is needed to further assess the technologies’ ability to improve UKA implant positioning, joint alignment, and reduce radiographic outliers for surgeons who do not perform a high volume of these operations. We conclude that in the hands of a high-volume UKA surgeon, NAV is equivalent to CI.

## Conflicts of interest

A. A. Sassoon is a paid consultant for Smith and Nephew, Biocomposites, and OrthAlign; receives research support from Biocomposites; receives other financial support from Smith and Nephew and Biocomposites; is an editorial board member of the Journal of Knee Surgery; and is a board or committee member of the American Association of Hip and Knee Surgeons. All other authors declare no potential conflicts of interest.

For full disclosure statements refer to https://doi.org/10.1016/j.artd.2023.101272.

## Author contributions

A.A.S. contributed to conceptualization, formal analysis, investigation, methodology, writing – review and editing. J.P. contributed to conceptualization, data curation, formal analysis, methodology, writing – original draft. C.J.M. contributed to formal analysis and methodology. J.T. contributed to conceptualization, data curation, formal analysis, investigation, methodology, writing – original draft, writing – review and editing.

## Appendix A Supplementary data

Conflict of Interest Statement for Mazzei

Conflict of Interest Statement for Sassoon

Conflict of Interest Statement for Patel

Conflict of Interest Statement for Taylor
