# Supplementary material for: A Comparative Evaluation of Conventional Instrumentation and Accelerometer-Based Navigation in the Practice of a High-Volume Unicompartmental Knee Arthroplasty (UKA) Surgeon
Source: Arthroplast Today. 2023 Nov 23;24:101272. doi: 10.1016/j.artd.2023.101272 (PMC10701135; doi:10.1016/j.artd.2023.101272)
Supplement: Conflict of Interest Statement for Sassoon [file mmc2.pdf]

INDIVIDUAL CONFLICT OF INTEREST STATEMENT

American Association of Hip and Knee Surgeons

(Adopted from the American Academy of Orthopaedic Surgeons disclosure statement)

The following form **must be filled out completely and submitted by each author (example, 6 authors, 6 forms). All items require a response. If there is no relevant disclosure for a given item, enter "*None.*"**

Manuscript Title: **A Comparative Evaluation of Conventional Instrumentation and Accelerometer-Based Navigation in the Practice of a High-Volume UKA Surgeon**

1. Royalties from a company or supplier (The following conflicts were disclosed): None

2. Speakers bureau/paid presentations for a company or supplier (The following conflicts were disclosed): None

3A. Paid employee for a company or supplier (The following conflicts were disclosed): None

3B. Paid consultant for a company or supplier (The following conflicts were disclosed): Smith and Nephew, Biocomposites, Orthalign

3C. Unpaid consultants for a company or supplier (The following conflicts were disclosed): Overture

4. Stock or stock options in a company or supplier (The following conflicts were disclosed): Orthalign

5. Research support from a company or supplier as a Principal Investigator (The following conflicts were disclosed): Biocomposties

6. Other financial or material support from a company or supplier (The following conflicts were disclosed):Fellowship Support: Smith and Nephew, Biocomposites

7. Royalties, financial or material support from publishers (The following conflicts were disclosed): None

8. Medical/Orthopaedic publications editorial/governing board (The following conflicts were disclosed): Journal of Knee Surgery (Associate Editor)

9. Board member/committee appointments for a society (The following conflicts were disclosed): AAHKS

In addition, one BLINDED Conflict of Interest form (no author names used) should be submitted per manuscript with all author disclosures.

ADAM SASSOON MD	[signature]	11/30/22
Author Name (Print or Type)	Author Signature	Date
